# Androgen Receptor Gene Variants in New Cases of Equine Androgen Insensitivity Syndrome

**DOI:** 10.3390/genes11010078

**Published:** 2020-01-10

**Authors:** Daniel A.F. Villagomez, Eastman G. Welsford, W. Allan King, Tamas Revay

**Affiliations:** 1 Department of Biomedical Sciences, University of Guelph, Guelph, ON N1G 2W1, Canadagwelsfor@uoguelph.ca (E.G.W.); waking@ovc.uoguelph.ca (W.A.K.); 2 Departamento de Produccion Animal, Universidad de Guadalajara, Zapopan 44100, Mexico; 3 Karyotekk Inc. Box 363 OVC, University of Guelph, Guelph, ON N1G 2W1, Canada

**Keywords:** androgen receptor, AR-gene mutation, androgen insensitivity, horse

## Abstract

In the domestic horse; failure of normal masculinization and virilization due to deficiency of androgenic action leads to a specific disorder of sexual development known as equine androgen insensitivity syndrome (AIS). Affected individuals appear to demonstrate an incoherency between their genetic sex and sexual phenotype; i.e., XY-sex chromosome constitution and female phenotypic appearance. AIS is well documented in humans. Here we report the finding of two novel genetic variants for the AR-gene identified in a Tennessee Walking Horse and a Thoroughbred horse mare; each in individual clinical cases of horse AIS syndrome.

## 1. Introduction

In mammals, androgenic actions are responsible not only for the normal differentiation of the male sex, but also for the development of the secondary sexual characteristics at puberty. The normal male ontogenesis sequentially includes the steps of testicular determination and sex differentiation, which is a complex process where endocrine testicular functions are crucial for the normal development of internal and external male sexual anatomy [1]. After testicular determination, steroidogenic testis cells, the Leydig cells are responsible for the production of androgens (e.g., testosterone hormone), which target specific tissues governing the mechanisms of masculinization and virilization, resulting in a normal male phenotype. Thus, the male internal system of sexual ducts ends with the formation of anatomical structures as, rete testis, efferent ducts, epididymis, deferent ducts, and accessory sexual glands, while androgenic action promotes the development of the external genitalia. On the other hand, virilization is a concept which refers to male germ cell differentiation, maturation, and the appearance of the secondary characteristics after puberty. 

In mammals, it is also known that a cytoplasmic protein receptor, the androgen receptor (AR), mediates those androgenic actions as a transcription factor of specific DNA sequences in the target tissues after translocating from the cytosol to the nucleus when bound to a testosterone metabolite such as dihydrotestosterone [2,3]. In this context, one may predict that damaging mutations in the AR-gene will induce either absence and/or malfunction of the corresponding protein receptor, prevent the target tissue from their normal sensitivity to androgens, and eventually lead to the disorder of sex development (e.g., the androgen insensitivity syndrome). In the domestic horse, there are only three previous reports documenting mutations of the AR-gene, causative for clinical cases of androgen insensitivity. In contrast, more than 1500 AR-gene variants have been documented in humans, as a genetic etiology for that type of syndrome [3]. Here, we report on two new equine AR-gene variants identified in horse mares showing the same type of disorder of sex development.

## 2. Materials and Methods

### 2.1. Clinical Cases

Case #1. A Tennessee Walking Horse with slightly ambiguous female-like external genitalia, increased anus-genital distance. Cranial cervix, vagina or uterus and intra-abdominal gonads were not detected. No estrous had been detected, but aggressive stallion-like behavior was observed. 

Case #2. A Thoroughbred horse mare with normal female external genitalia, but had short blind-ending vaginal vault, without a cervix or uterus. Intra-abdominal testes-like structures were observed on ultrasound.

These two animals were submitted by the owners for a routine reproductive assessment at the MH Gluck Equine Research Center, University of Kentucky. Blood samples were collected by veterinarians according to the Center’s best practice guidelines. Leftover DNA samples were then provided by the laboratory of the late Dr. Teri L. Lear [4] and were used according to the University of Guelph’s Animal Care Committee guidelines.

No other family members from either case were available for testing.

### 2.2. Cytogenetics and Sexing

Trypsin-Giemsa banded (GTG) karyotypes were arranged from the clinical cases following recommendations of the International system for cytogenetic nomenclature of the domestic horse [5]. A conventional cytogenetics protocol as previously described [6] from routine peripheral lymphocyte cultures revealed a 2*n* = 64, XY chromosomal constitution in both animals. 

The presence of the Y-chromosomal SRY gene was detected in both animals by PCR according to Paria et al. [7], and Bolzon et al. [8].

### 2.3. Sequencing the AR Gene

Sequencing the whole coding portion and exon–intron junctions of the equine AR gene was done by Sanger sequencing using eleven primer pairs (E1B,C,D,E-F/R and E2,3,4,5,6,7,8-F/R), as described by Revay [9]. In brief, the PCR mixture of 20 ng DNA, 0.5 μM primers, and 1× AmpliTaq Gold 360 master mix (Thermo Fisher Scientific, Waltham, MA, USA) was amplified in an MJ Research PTC-200 Thermo Cycler (Bio-Rad, Hercules, CA, USA) with the following thermal profile: 95 °C for 10 min and 30 cycles of 94 °C, 45 s; 59 °C, 45 s; 72 °C, 45 s, and a final elongation at 72 °C for 10 min. Sequencing of the visualized PCR products was conducted using fluorescent dye-terminator cycle sequencing and capillary electrophoresis at the Laboratory Services Division of the University of Guelph. The sequences were checked for quality and aligned to the GenBank horse AR reference sequence (NC_009175.3, NM_001163891.1) using Geneious Pro software v.7.1.7 (Geneious Inc., San Diego, CA, USA). 

Sequences were deposited to the NCBI GenBank (submission ID: 2283909).

### 2.4. Sequence Variant Analysis

Variants were named according to the Human Genome Variation Society (HGVS) recommendations [10]. Variants were mapped to the horse AR gene at the DNA and protein levels, then classified using a comparative approach where the limited information available for the biology, function and genetic variations of the horse AR was supplemented with the available literature and disease associations for the human ortholog. Described sequence variants were searched within the following databases: the Online Mendelian Inheritance in Animals (OMIA, https://omia.org/OMIA000991/9796) the Androgen Receptor Gene Mutations Database (ARDB, http://androgendb.mcgill.ca) [3], the Leiden Open Variation Database (LOVD, https://databases.lovd.nl/shared/transcripts/AR), ClinVar (https://www.ncbi.nlm.nih.gov/clinvar) [11], and gnomAD (https://gnomad.broadinstitute.org) [12]. UniProt (https://www.uniprot.org) and Varsome (https://varsome.com) was used to help classify the variants. 

## 3. Results

Both animals investigated here presented with the clinical phenotype of disorders of sexual development, with infertility, female-like external genitalia, and hypoplastic internal reproductive organs. As the chromosomal sex identified as male with the corresponding 64, XY karyotype with the presence of SRY gene, a 64, XY DSD syndrome and Androgen Insensitivity Syndrome was suspected, leading to the molecular testing of the AR-gene. 

**Figure 1:**
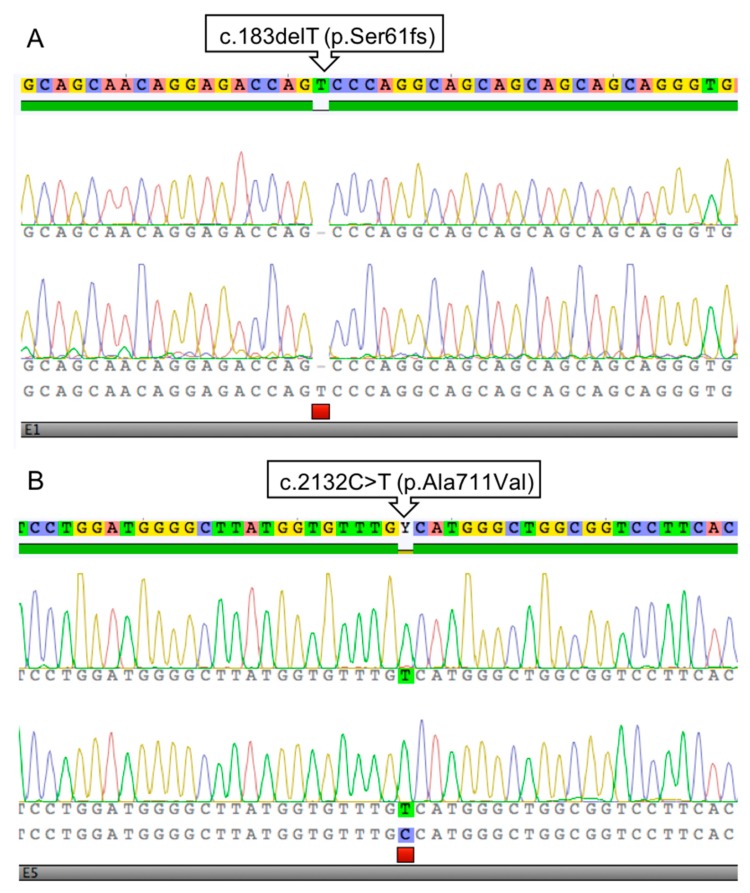
Sanger sequencing electrophoretograms presenting the c.183delT (p.Ser61fs) variant in Case #1 (**A**) and c.2132C>T p.Ala711Val) in Case #2 (**B**).

Sequencing resulted in the identification of two distinct sequence variants, c.183delT in Case #1 and c.2132C>T in Case #2 (Figure 1).

The single nucleotide deletion identified in Case #1 is situated in exon 1 (NM_001163891.1:c.183delT) and it alters the Serine coding (AGT) codon to (AG-). This frame shift predictably leads to a very short, truncated protein when extended until a premature STOP codon 89 amino acid residues downstream. The variant can be described at the protein level as NP_001157363.1:p.(Ser61SerfsTer89) or in short as NP_001157363.1:p.(Ser61fs). In agreement with the XY chromosome constitution, the animal is hemizygous for the variant (Figure 1A).

In Case #2, a different sequence variant was identified in exon 5 (NM_001163891.1:c.2132C>T) leading to a codon change (GCC)>(GTC), thus it is a missense change from Alanine to Valine (NP_001157363.1:p.(Ala711Val), Figure 1B).

## 4. Discussion

In the past, the inconsistency observed in mammalian species between the chromosomal sex and phenotypic sex (i.e., XX males and/or XY females) was called sex reversal syndrome. Furthermore, intersexuality was also widely used to describe a broad range of pathologies related to abnormal sex determination, as ambiguous gonadal sex as well as sexual phenotypic ambiguity. Following the adoption of the consensus nomenclature in human medicine, pathologies involving the alteration of the normal definition of components of sex development are referred to as disorders of sex development or DSDs [13]. In humans, one of the most prevalent DSDs reported in the literature is the Androgen Insensitivity Syndrome. More than 1500 genetic variants for the AR-gene have been identified as the genetic etiology for that pathology. Affected individuals lack a correspondence between their genetic sex (e.g., XY sex chromosome constitution), and the expected phenotypic sex, so that they may show a female phenotype and/or a range of sexual ambiguity. This pathology manifests as a consequence of deficient androgen actions (e.g., androgen insensitivity) in target tissues during normal male sex ontogenesis. In the domestic horse, we previously described the occurrence of the androgen insensitivity syndrome, where affected individuals demonstrated a female-like phenotype but having a normal stallion karyotype [6]. The equine androgen insensitivity syndrome was also proposed as a genetic etiology due to association of familial sequence variants in the AR-gene segregating among carrier mares and affected stallions [8,9,14]. 

**Figure 2:**
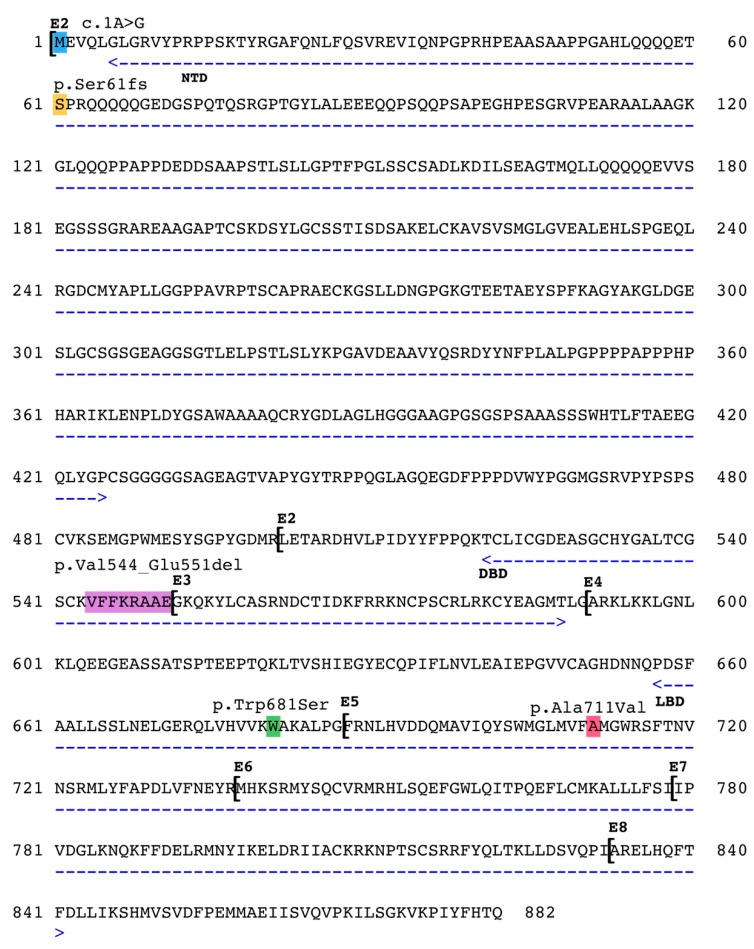
Pathogenic variations in the horse AR. Functional domains are marked with arrows, NTD = N-terminal Domain, DBD = DNA Binding Domain, LBD = Ligand Binding Domain. Exons marked with E1-E8. c.1A>G by Revay et al., 2012 [9], p.Val544_Glu551del by Welsford et al., 2017 [14], p.Trp681Ser by Bolzon et al., 2016 [8]. The p.Ser61fs and the p.Ala711Val variants are described in this paper.

Thus, while there are more than 1500 variants in the human androgen receptor gene [3] only three different mutations in the horse AR gene are currently listed in the Online Mendelian Inheritance in Animals database and are thought to be causative of equine AIS (OMIA# 000991–9796, https://omia.org). These variants are located in different and critical parts of the gene, as depicted in Figure 2. A very rare type of pathogenic variant in the start codon (c.1A>G) was shown to affect the expression of the AR protein in a family of American Quarter horses [9]. A missense variant in exon 4 (c.2042G>C) resulting in the exchange of a highly conserved tryptophan residue to a serine (p.Trp681Ser) in the Ligand Binding Domain segregated among affected and normal members of a Thoroughbred family [8]. Finally, the 25 nt long deletion in AR exon 2 of Warmblood horses (c.1630_1654del) was predicted to cause truncation of the protein and/or detrimental changes of the DNA binding capacity of the receptor [14].

Here we report two novel sequence variants in the horse AR. The first in Case #1 is a single nucleotide deletion (c.183delT), resulting in a frame shift and a predicted truncation at a premature stop site downstream in exon 1 (at position 452 nt). This region in exon 1 encodes for the N-terminal domain (NTD) of the 95 kDa sized androgen receptor that modulates the protein function through co-regulator binding, transactivation units and phosphorylation sites [15]. Interestingly, the deletion localizes to one of the several serine phosphorylation sites of the AR. The mutation affects Ser61 of the horse AR, that is conserved in the human AR (Ser83). Note that earlier publications used an older protein nomenclature and identified this same site as Ser81 (see note at ARDB, www.androgendb.mcgill.ca). Moreover, Ser83 is the highest stochiometric phosphorylation site in the human AR, that greatly affects the receptor function [16]. It was also shown that CDK5 and CDK9 kinases specifically act on this residue and regulate AR chromatin binding [17] and the transcription of AR target genes [18]. Additional to the functional importance of this locus, a human case affecting the same region by a 10 nt long deletion was described by Audi et al. [19]. That variant leads to the same frame shift and predicted truncation, as in our case. The two affected individuals presented with complete androgen insensitivity phenotype and the complete lack of substrate binding activity of the AR was shown in laboratory tests. The variant was not identified in any human reference populations in gnomAD. Based on these results the c.183delT nonsense variant can be classified as pathogenic.

The missense variant identified in Case #2 is situated in exon 5 that is part of the Ligand Binding Domain (LBD) encoded by exons 4–8. This highly conserved protein domain is involved in androgen binding, activation, and dimerization; all critical functions of the androgen receptor [15]. It is not surprising that the largest number of missense variants—identified in human cases—accumulate in this domain. Among the more than 600 missense variants in the LBD there are two that alter the same conserved Alanine codon (A749), as observed in our case. Thermolability and abnormal ligand dissociation kinetics were measured in two cases of AIS when the same Alanine changed to Aspartic acid [20,21]. The same Alanine to Valine change (p.A749V) was described as a somatic variant in prostate cancer and estimated to have an adverse effect on the androgen receptor function [22]. Moreover, according to the Varsome classification software, nine in silico prediction algorithms—including SIFT and MutationTaster—classify this missense change as damaging/disease causing. Thus, the cumulative human data strongly support the conclusion that the p.A711V horse AR-gene variant causes the observed pathogenicity 

In summary, the two unique AR-gene allelic variants identified here could be classified as pathogenic and likely responsible for the observed AIS phenotype in the investigated animals.
